# Development and validation of a photographic atlas of food portions for accurate quantification of dietary intakes in China

**DOI:** 10.1111/jhn.12844

**Published:** 2021-01-06

**Authors:** Ye Ding, Yue Yang, Fang Li, Yingying Shao, Zhongqing Sun, Chunmei Zhong, Ping Fan, Zuwen Li, Man Zhang, Xiaocheng Li, Tingting Jiang, Chenglin Song, Dandan Chen, Xiaoju Peng, Lu Yin, Yuanhong She, Zhixu Wang

**Affiliations:** ^1^ Department of Maternal, Child and Adolescent Health School of Public Health Nanjing Medical University Nanjing China; ^2^ Sir Run Run Hospital Nanjing China; ^3^ Qingdao Municipal Center for Disease Control & Prevention Qingdao China; ^4^ Department of Nutrition The Affiliated Hospital of Qingdao University Qingdao China; ^5^ Changzhou Center for Disease Control and Prevention Changzhou China; ^6^ Kun Shan Market Regulatory Administration Suzhou China; ^7^ Department of Nutrition and Food Hygiene School of Public Health Peking University Beijing China; ^8^ Nanjing Municipal Center for Disease Control and Prevention Nanjing China; ^9^ Nanjing Brain Hospital Nanjing China; ^10^ The Second People's Hospital of Lianyungang Lianyungang China; ^11^ Huai'an Maternal and Child Health Center Huai'an China; ^12^ Suzhou Maternal and Child Health Care & Family Planning Service Center Suzhou China

**Keywords:** food atlas, visual reference systems, food estimation, retrospective dietary survey

## Abstract

**Background:**

Accurate estimation of food portion sizes remains an important challenge in dietary data collection. The present study aimed to develop a food atlas with adequate visual reference to improve the accuracy of dietary surveys in China.

**Methods:**

A food atlas for dietary surveys in China was developed using three visual reference systems, namely, regularly placed food portions, the two‐dimensional background coordinates and common objects known in daily life. The atlas was validated by estimating a meal before and after using the food atlas, and differences in weight estimation were compared using a paired *t*‐test. In total, 50 college students participated in the study.

**Results:**

After determination of food varieties; design of the food display; purchase, processing, cooking and weighing of food; photographing food; post‐image processing and data processing, a total of 799 pictures of 303 types of food and two types of tableware were produced. The mean value of food weight estimated with the atlas was closer to the actual weight, and the variation range of these values was smaller and more stable than that estimated without the atlas. The differences estimated before and after using the atlas for all foods were significant (*P* < 0.05). Comparing the differences in weight before using the atlas, the error ranges of food samples were reduced.

**Conclusions:**

A food atlas has been developed for a retrospective dietary survey in China, which can be used to enable a better understanding of nutritional adequacy in the Chinese population.

## Graphical abstract

Accurate estimation of food portion sizes remains an important challenge in dietary data collection. We developed a food atlas for retrospective dietary surveys in China, which can be used to enable a better understanding of nutritional adequacy in the Chinese population.

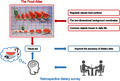

## INTRODUCTION

Dietary data facilitate the understanding of the food and nutrient intake of a population or an individual within a certain period. It is necessary to collect accurate dietary intake data to provide information for national and global health and nutrition policies^1^, ^2^. Currently, the most commonly used method to obtain dietary information is the 24‐h recall method^3^, ^4^. This method relies on the respondents’ recall and description to quantify their past food intake^5^. The longer the time interval between the dietary review and the meal, the greater the error and lower the accuracy of the data. Furthermore, this retrospective method is often based on respondents’ assessment of food and requires respondents to have a certain level of education and life experience in practical work. However, in the thinking concept of general population, there is a lack of relationship between the visual impression of the appearance of all types of food and the weight of the corresponding food; therefore, the accurate estimation of food weight remains an important challenge in dietary data collection^6^, ^7^. Both under‐ and over‐reporting of food consumed are major sources of error in the assessment of dietary structure, as well as energy and nutrient intake. Therefore, the need for visual aids in surveys is urgent; it can help respondents remember and accurately describe the food amount. Moreover, several food atlases are available, and the validity of a food atlas as a tool in dietary data collection has been studied globally, supporting its usefulness in quantifying food portions^8^, ^9^, ^10^, ^11^, ^12^, ^13^, ^14^, ^15^. However, there are few food atlases with sufficient visual reference systems for retrospective dietary surveys in China. Because food habits vary worldwide, it is essential for dietary surveys to use a food atlas that is appropriate to the local context.

Rapid economic growth and urbanisation have brought remarkable changes in China, impacting diet and leading to an increasing prevalence of overweight and obese individuals, as well as diet‐related non‐communicable diseases^16^, ^17^, ^18^, ^19^. Thus, locally relevant dietary assessment tools for determining the dietary structure and energy and nutrient intake of the Chinese population are essential^20^, ^21^. Many auxiliary tools have been used in dietary surveys in China, including tableware (bowls, cups, spoons, etc.) for calibrating food weight (capacity), measuring tools (salt spoons, oil cups, etc.) and food models^22^, ^23^. However, as a result of the numerous Chinese food items, the processing and cooking methods are complex and diverse^24^, ^25^, and the types of auxiliary tools are very limited and have considerable limitations in their application. Specifically, the tableware and measuring tools used for calibrating food weight or capacity have their own suitable food types. For example, a salt spoon is suitable for measuring salt, monosodium glutamate and other condiments; an oil pot is suitable for measuring vegetable oil and other oils. Food models play an important role in clinical diet guidance for patients with chronic non‐communicable diseases, but the existing food models are relatively limited for the general population. In other words, the coverage of food varieties is insufficient. If the variety of food models is expanded, it will cost more and cannot be used in field investigation because of the inconvenience of carrying^23^. Therefore, none of them are suitable for large‐scale on‐the‐spot investigations. Food pictures are dietary survey tools that have been developed in recent years, typically containing many different types of food. They are portable and easy to carry for field research. However, because of the differences between picture vision and food vision, there is a lack of general food pictures with adequate visual reference in China. Accordingly, it is difficult to help respondents effectively determine the weight of food. For example, quantitative pictures of some foods were provided in the *China Food Composition Tables 2009* ^26^, but no reference system for these pictures existed. In food pictures without a reference system, no difference between a 25‐g and a 200‐g orange or a 25‐g flour steamed bread and a 100‐g flour steamed bread may exist, and a 40‐g egg can appear similar to an 80‐g egg. These discrepancies are a result of differences in imaging distance.

To overcome these limitations in practical work, our research group used three visual reference systems: regularly placed food portions, the two‐dimensional background coordinates and common objects known in daily life, and consequently developed a food atlas for retrospective dietary surveys in China, which can help respondents and investigators better estimate the amount of food consumed according to the visual information of food in their memory.

## MATERIALS AND METHODS

### Design and production of the food atlas

#### Determination of food varieties and status, as well as processing and cooking methods

China has a vast and varied territory with abundant food ingredients. As a result of cost and time constraints, it was necessary to preselect the foods for inclusion in the atlas. From the perspective of auxiliary estimation of food quantity, food with the same appearance, shape and density can be supported by the same food picture. For example, the appearance of thin pork, beef and mutton is relatively consistent. Thus, after the classification and summary of ordinary food with the same or similar appearances, we made pictures of >300 types of food, including some food with Chinese characteristics, such as Flos Sophorae (Chinese name: *yang huai hua*; flowers and buds of *Sophora japonica*), Chinese toons (Chinese name: *xiang chun*; Sapindales, Meliaceae; tender bud), bean curd, pig intestine and freshwater fish, etc., all of which were displayed in detail.

There are various Chinese processing and cooking methods that can affect the visual impression of food. For example, for similar weighing potatoes, the amount of shredded potatoes appears to be greater than the amount of potato chips. Therefore, we considered the different statuses of food, different processing and cooking methods. Processing information about vegetable included whether it was in the original state; peeled, cut into chunks, pieces and segments; and shredded thinly. Meanwhile, cooking information included whether it was boiled, blanched, stir‐fried, burnt, braised, fried, steamed or stewed, etc. For example, rice was boiled in water, vegetables were blanched in boiling water and pork was stir‐fried with vegetable oil.

#### Weighing of food samples to obtain the percentage of edible portion

After dietary data collection, intake of various foods was obtained to evaluate the dietary structure, as well as the energy and nutrient intake. The intake of food refers to the weight of all types of food that can be 100% consumed in its classical state. For example, fruits and vegetables are 100% edible weight in their fresh state, and cereals and their products are 100% edible weight in their dry state. Therefore, it is important to accurately obtain information on food intake. To achieve this goal, the food was weighed throughout the process of food selection, processing and cooking, so that the atlas could provide the percentage of edible parts, raw/cooked ratio, dry/wet ratio and other information with respect to its role in the retrospective dietary investigation. The UWA‐K electronic scale (Beijing Lianchang Jiaxu Electronic Technology Co., Ltd, Beijing, China) was used to measure the weight of food samples, with an accuracy of 0.1 g and prior calibration.

#### Selection of food samples and determination of portion sizes

First, a certain food was selected and then, according to its size or quantity, it was set as a certain number of food portions based on its natural form or within the range of the most common consumption quantity. According to different types of food, four to 10 different grades of food portions were designed. Specially, for a certain single or single large food, the whole or part of the single food was selected as a certain amount of food portion; for a medium‐sized foods or foods processed and cooked or partially processed, a certain amount of them was selected as a certain quantity grade of food portion.

#### Sizing reference and display of items

The first visual reference was the food itself. The food portions were displayed from left to right in the order of quantity from less to more, or size from small to large, or weight from light to heavy. The second visual reference was the background coordinates. According to its size or weight, the food portions of each grade (measured using the corresponding weight) were regularly displayed on the background of vertical and horizontal coordinates calibrated in units of length (scale with 1 cm × 1 cm). To better set off the food itself and achieve the best visual effect of food pictures, the background had two colours: black and white. When dark‐coloured foods (such as green vegetables) were photographed, the white background was selected, whereas the black background was mainly used for photographing light‐coloured foods (such as wheat and its products), aiming to ensure a sharp contrast between the background and the colour of the food itself. Furthermore, the background was placed in the form of an inverse parabola, which can make the food picture appear three‐dimensional. To obtain a better visual reference, common objects known in daily life were placed beside or in the middle of the food portions as the third visual reference of shape and size, mainly including a 355‐mL aluminum can and a piece of paper‐packed gum.

Fresh and unprocessed food ingredients were photographed directly on the background, whereas some processed and cooked foods were placed on common household tableware such as plates, bowls or spoons. In Chinese tableware, plates are most commonly employed and used to hold food other than the staple food. Plates of different colours and sizes were selected according to the colour, shape and quantity of food, including round purple plates (diameter = 17 cm), round orange plates (diameter = 17 cm or 20 cm), round white plates (diameter = 12 cm), long white plates (length = 17 cm, width = 8 cm or length = 19 cm, width = 12 cm), plates with purple edges (diameter = 15 cm or 20 cm) and lace plates (diameter = 15 cm or 20 cm). Most Chinese staple foods (such as rice and porridge) are contained in bowls. The glass bowls used in this atlas had eight sizes (6, 9, 10, 11, 12, 13, 14 and 17 cm in diameter, respectively). Furthermore, information regarding some condiments (such as salt, monosodium glutamate, white sugar, etc.) was displayed with a spoon, and the amount of liquid food (such as water, beverage, milk) with different volumes was measured using glasses (diameter = 6, 7 or 8 cm).

#### Photographic records of food pictures

The visual relationship of these elements (regularly placed food portions, background coordinates, and reference objects) was photographed using a camera (DX AF‐S Nikkor 18–70 mm; Nikon, Tokyo, Japan) under the same conditions: shooting angle, 45°; distance from the vertical and horizontal coordinate background, 55 cm; aperture, f/20; focal length, 70 mm. All photographs were taken at a scale of 16:9, with a 6–7 MB photo size (3000 × 4000 pixels). Then, according to the result of the previous weighing, the weight information of the food portions was marked in the photograph to make a food picture. To obtain the most realistic pictures possible, the images were colour‐printed with dimensions of 75 × 100 mm.

#### Food pictures collection to form food atlas

The pictures of all types of food in different states and tableware were summarised and classified into 12 categories, including (1) cereals and its products, potatoes, and beans excluding soybeans; (2) vegetables; (3) fruits; (4) livestock and poultry meat; (5) fish, shrimp and shellfish; (6) eggs; (7) soybeans and soybean products; (8) nuts; (9) cooking oils; (10) cakes, candies and condiments; (11) compound processed foods; and (12) tableware. Then, they were combined with the relevant food information, such as the percentage of edible parts, raw/cooked weight ratio and dry/wet weight ratio, as well as the relevant food concepts and information about energy and nutrients in food. Finally, the pictures were compiled into a book, forming an auxiliary reference food atlas to be used for retrospective dietary surveys.

### Validation of the food atlas

#### Implementation of the study

An observational study was conducted to validate the food atlas. Fifty college students from Nanjing Medical University, aged 20–22 years old, were invited to participate. The project was approved by the Human Research Ethics Committee of Nanjing Medical University (Protocol # 33; 2014/2015). Oral and written informed consent were obtained from each participant.

After selecting 10 types of food with Chinese characteristics, a meal was prepared according to the common processing and cooking methods. The investigators were responsible for weighing and calculating the raw food. The main ingredients included rice, tomato, celery, mushroom, rape (scientific name: *Brassica napus* L.; Chinese name: *you cai*; a green leafy vegetable), pork, crucian carp (scientific name: *Carassius auratus*; Chinese name: *ji yu*; one of the most common freshwater fish in China), egg, orange and banana. The investigators put the food in bowls or plates, and participants estimated the weight of the main ingredients. Then, the investigators put the bowl or plate containing the food on the background of vertical and horizontal coordinates calibrated in units of length (scale with 1 cm × 1 cm), next to the common objects known in daily life (a 355‐mL aluminum can and a piece of paper‐packed gum). The participants then estimated the weight of the main ingredients again according to the pictures of the relevant ingredients in the auxiliary reference food atlas. Each person estimated the weight of a food twice, with and without the use of the auxiliary reference food atlas. The differences between the estimated weight and the actual weight were then calculated and compared in the subsequent analysis of the data.

#### Quality control

All of the investigators underwent strict training and assessment in quality control procedures to ensure the quality in their work. Before the implementation of the study, a preliminary investigation was conducted, and strict quality control was conducted. Furthermore, investigators communicated relevant procedures to study participants to promote their cooperation.

#### Statistical analysis

The relative difference (*d*) was calculated as: *d *= estimated weight − actual weight. Meanwhile, the absolute difference (*D*) was calculated as: *D* = |estimated weight − actual weight|. Then, the percentage of *d* and *D* in the actual weight was calculated using the formulae:

d%=[(estimated weight‐actual weight)/real weight]×100

D%=(|estimated weight‐actual weight|/real weight)×100

For each food, the differences in weight estimation with and without the help of the auxiliary reference food atlas were compared using a paired *t*‐test. Statistical analysis was performed using spss, version 20.0 (IBM Corp., Armonk, NY, USA). *P* < 0.05 was considered statistically significant.

## RESULTS

### Overview of the food atlas

After selection and determination of food varieties; design of the food display; purchase, processing, cooking and weighing of food; photographing food; post‐image processing and data processing, a total of 799 pictures of 303 types of food and two types of tableware with three visual reference systems were produced. At the same time, the percentage of edible parts, raw/cooked weight ratio and dry/wet weight ratio, as well as the relevant food concepts and information about energy and nutrients in food, were also completed.

All food pictures were classified into 12 categories. The classification and number of pictures are shown in Table 1.

**Table 1:** Type and number of foods and tableware included in the food atlas

Category	Food item	Food	Food varieties	Total number (food or tableware)	Food photos	Total number (picture)
1	Cereals and its products, potatoes, and beans excluding soybeans	Wheat and its products	11	26	31	67
		Rice and rice products	3		8	
		Food grains other than wheat and rice	6		14	
		Beans excluding soybeans	1		3	
		Potatoes	1		3	
		Noodles made from bean or sweet potato starch	4		8	
2	Vegetables	Vegetables with edible parts of stems, leaves, stalks and flowers	25	62	116	267
		Vegetables with edible parts of rhizomes	11		45	
		Cucurbitaceous vegetables	8		39	
		Solanaceous vegetables	2		6	
		Leguminous vegetables and sprout	10		32	
		Fungi and algae	5		28	
		Paprika	1		1	
3	Fruits	Pome fruits	8	49	7	84
		Stone fruits	14		27	
		Citrus fruits	3		5	
		Melon fruits	6		16	
		Berry fruits	9		16	
		Tropical fruits	9		13	
4	Livestock and poultry meat	Livestock meat and their internal organs	19	28	37	65
		Poultry meat and their internal organs	9		28	
5	Fish, shrimp and shellfish	Freshwater fish	18	74	37	196
		Marine fish	30		72	
		Shrimp and crab	14		35	
		Shellfish	12		52	
6	Eggs	–	3	3	6	6
7	Soybeans and soybean products	Soybeans	2	8	5	18
		Soybean products	6		13	
8	Nuts	Nuts of herbaceous plants	7	13	16	28
		Nuts of woody plants	6		12	
9	Cooking oils	–	1	1	1	1
10	Cakes, candies and condiments	Cakes	19	29	20	34
		Candies	8		10	
		Condiments	2		4	
11	Compound processed foods	–	10	10	28	28
12	Tableware	–	2	2	5	5

### Example extracts from the food atlas

#### Cereals, potatoes and beans excluding soybeans

**Figure 1:**
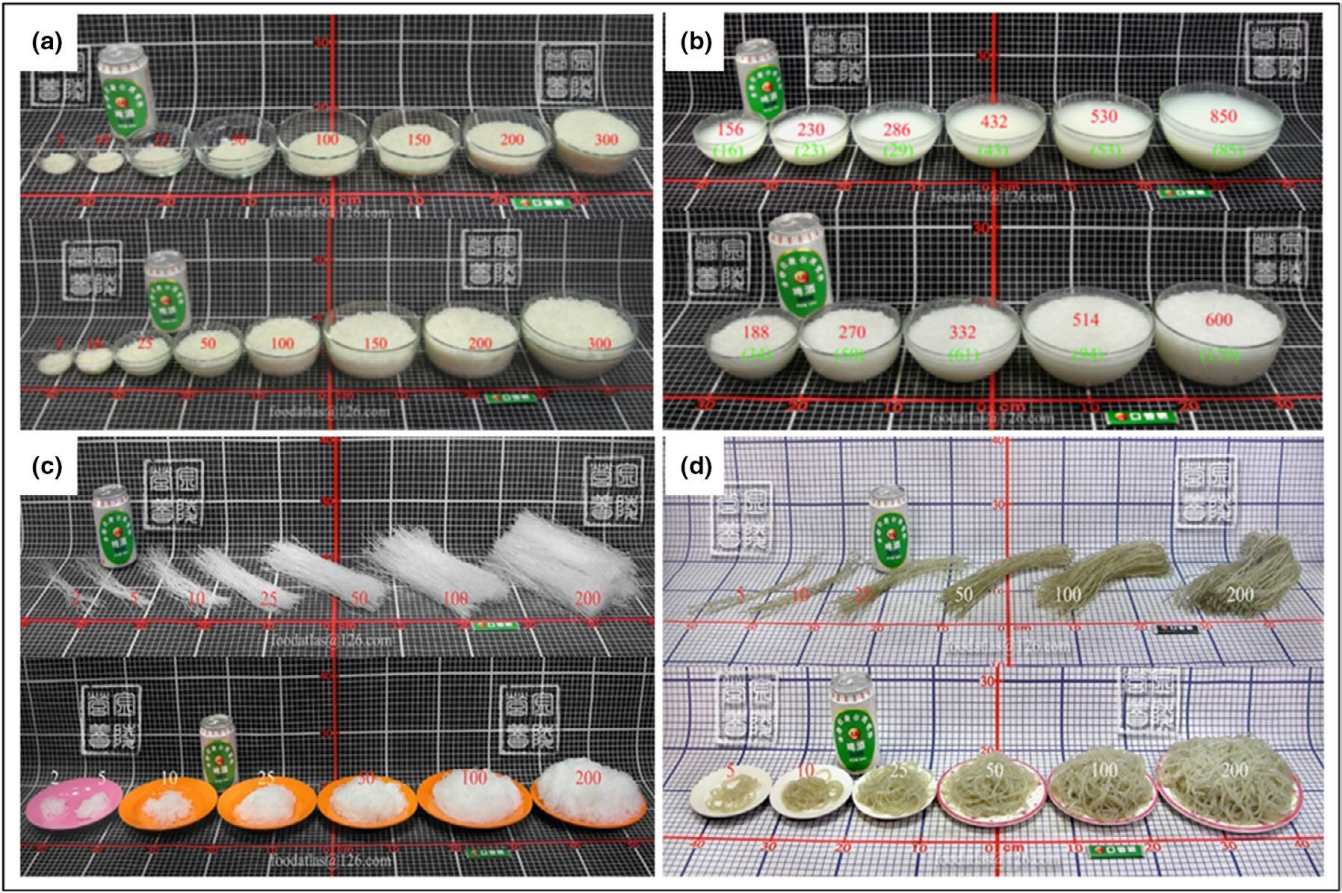
Photos of rice and vermicelli in different states. (a) Raw and dry rice (above) and steamed rice (below). (b) Sparse rice porridge (above) and thick rice porridge (below). (c) Raw (above) and cooked (below)Vermicelli made of starch from beans excluding soybeans. (d) Raw (above) and cooked (below)Vermicelli made of starch from potatoes.

The main forms of this category of food displayed in the atlas include wheat and its products, rice and its products, millet, corn, oats, mung beans and potatoes, etc. As a result of the complexity and diversity of processing and cooking methods, this category of food can take a variety of forms (e.g. rice and vermicelli). The presentation of rice includes raw and dry rice in a bowl, as well as sparse and thick rice porridge (Figure 1a,b). Vermicelli is made of starch from potatoes or beans excluding soybeans. The atlas shows the original state of vermicelli from different sources and the state of water removal after cooking (Figure 1c,d).

#### Vegetables

**Figure 2:**
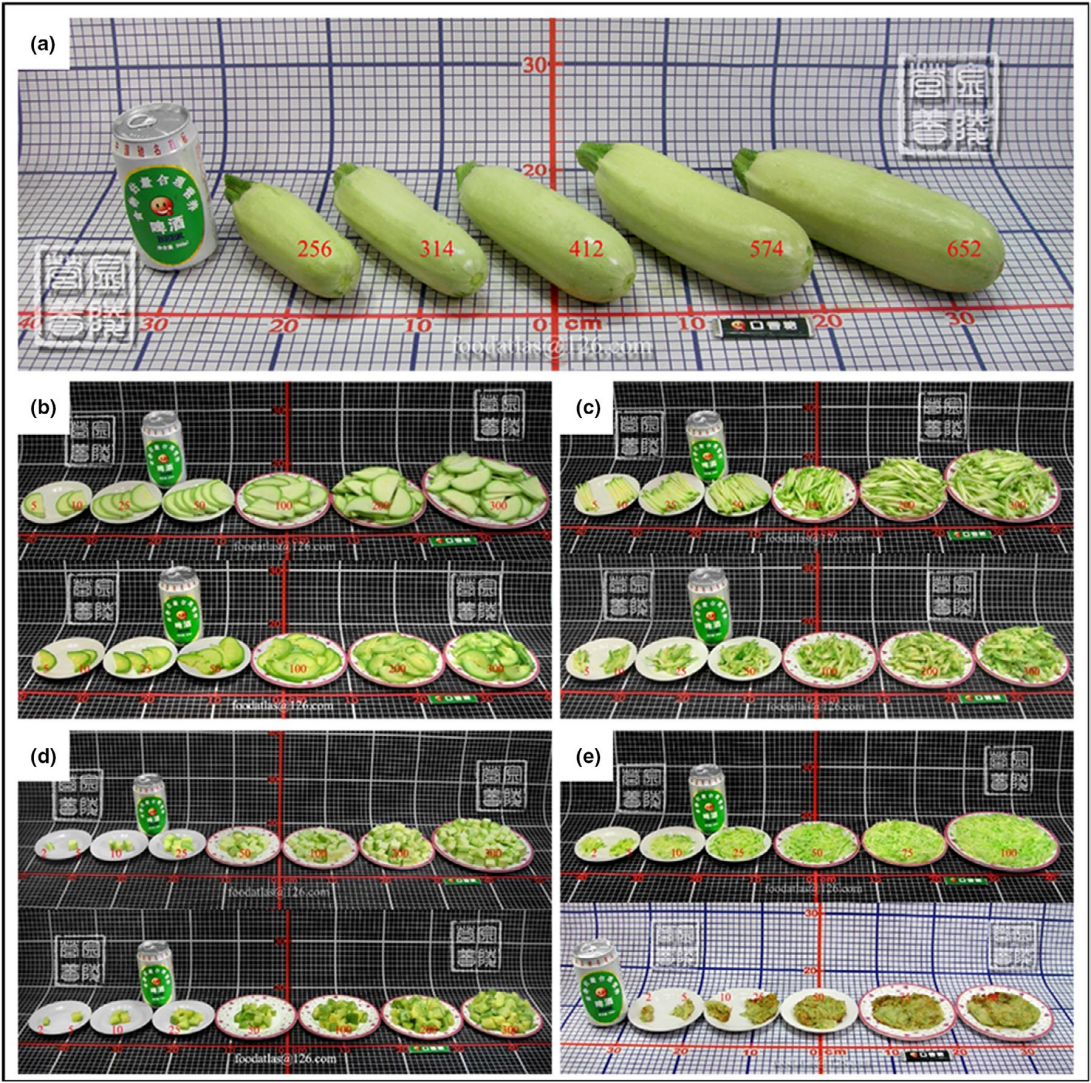
Photos of zucchini in different processing methods. (a) The whole zucchini. (b) Raw (above) and cooked (below) zucchini cut into pieces. (c) Raw (above) and cooked (below) zucchini cut into strips. (d) Raw (above) and cooked (below) diced zucchini. (e) Raw (above) and cooked (below) shredded zucchini.

Vegetables are displayed as a whole, in different parts (such as leaves and stalks), after undergoing different processing methods (such as slicing and shredding). Taking zucchini as an example, the pictures show the different states of the vegetable, such as the whole zucchini, as well as when it is cut into pieces and strips, diced and shredded (Figure 2). The fresh weight of zucchini is shown. Except for the whole zucchini, the other forms are 100% edible.

#### Fruits

Each fruit is first shown in its original state according to different sizes or quantities. It is then displayed separately by peeling, removing the core, slicing, dicing and other different processing methods. Taking Hami melon as an example (see Supporting information, Figure S1), the fresh weight in different states is shown, and the blue number is the weight in a 100% edible state.

#### Livestock and poultry meat

**Figure 3:**
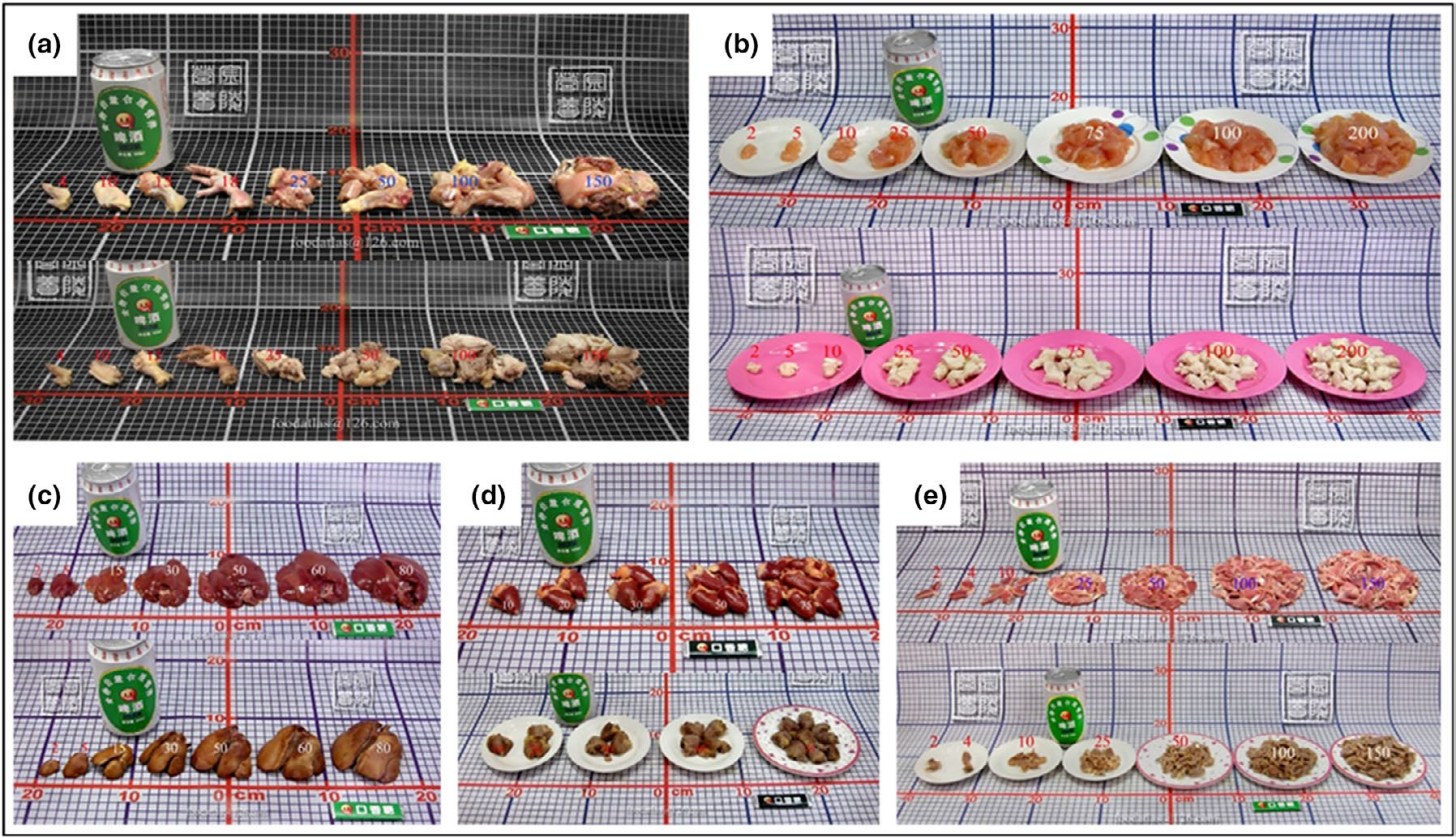
Photos of raw (above) and cooked (below) chicken and internal organs of chicken. (a) The whole chicken were divided into different parts. (b) Different portions of chicken breast meat. (c) Chicken liver. (d) Chicken heart. (e) Chicken gizzard.

The livestock and poultry meat in this atlas mainly comprise livestock meat, poultry meat and their internal organs. They are displayed on plates in raw and cooked states. Taking chicken as an example, the weight of the whole chicken divided into different parts and the different portions of chicken breast meat are shown in Figure 3a,b, and the internal organs of chicken are shown in Figure 3c,d,e.

#### Fish, shrimp and shellfish

**Figure 4:**
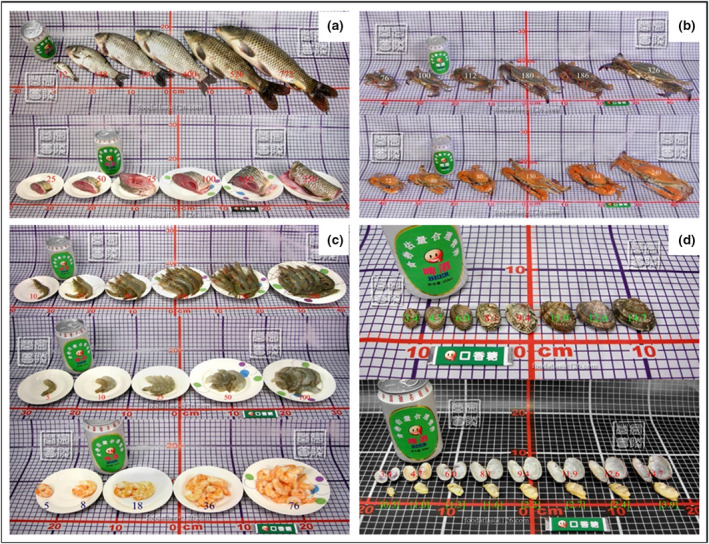
Photos of fish, shrimp, crabs and shellfish. (a) Crucian and carp displayed in whole (above) or in part (below) by size or weight. (b) Raw (above) and cooked (below) swimming crabs. (c) The marine shrimps with shells, shelled fresh shrimps and cooked shrimps. (d) Clams with shell and separated shell and meat.

Fish, shrimp and shellfish are divided into freshwater fish, sea fish, shrimp, crabs and shellfish. Each fish is displayed in whole or in part by size or weight. Figure 4a depicts an example of a crucian and carp. Crabs are displayed according to their size in raw and cooked states, and Figure 4b shows an example of swimming crabs. The shrimp and shellfish are displayed according to whether they had shells or not. The marine shrimps with shells, shelled fresh shrimps and cooked shrimps are shown in Figure 4c from top to bottom. Clams with shell and separated shells and meat are shown in Figure 4d.

#### Eggs

Eggs mainly include quail, chicken, duck, and goose eggs. The atlas includes various existing forms of eggs, such as fresh state, fried eggs, scrambled eggs and egg yolk. The atlas shows the relationship between visual images of various states of eggs and weight, including eggs in the shell, poached eggs, scrambled eggs and full egg yolks. Some forms of eggs are shown in the Supporting information (Figure S2).

#### Soybeans and soybean products

Soybeans and soybean products are mainly divided into raw soybeans and their products. Soybean products include bean curd, skin of soybean milk, dried bean curd and yuba, etc. Different cooking methods such as soaking and stewing in soy sauce are considered. An example of soybeans and bean curd is provided in the Supporting information (Figure S3).

#### Nuts

The nuts shown in the atlas are mainly from herbaceous (peanuts, sunflower seeds, etc.) and woody plants (walnuts, chestnuts, etc.). Each type of nut is stacked and displayed in two states: with and without shells. Taking peanuts and walnuts as an example, the pictures show different states (see Supporting information, Figure S4).

#### Cooking oils

The cooking oil in the atlas comprises chilli oil put in small bowls (see Supporting information, Figure S5).

#### Cakes, candies and condiments

There are many types of cakes in the atlas, involving many cooking methods, such as steaming, baking and frying, etc. Cakes, biscuits, walnut crisp cakes, fried dough twists and mooncakes are displayed according to their different shapes and sizes. An example of biscuits and mooncakes is provided in the Supporting information (Figure S6). Candies in the atlas include lollipops, fruit flavored hard candy, butterscotch, nougat and chocolate, etc. Different shapes and sizes of chocolates are shown in the Supporting information (Figure S7). The condiments on display are white sugar, iodised salt and sweet sauce from top to bottom (see Supporting information, Figure S8) and different portions of them are displayed in different tableware.

#### Compound processed foods

**Figure 5:**
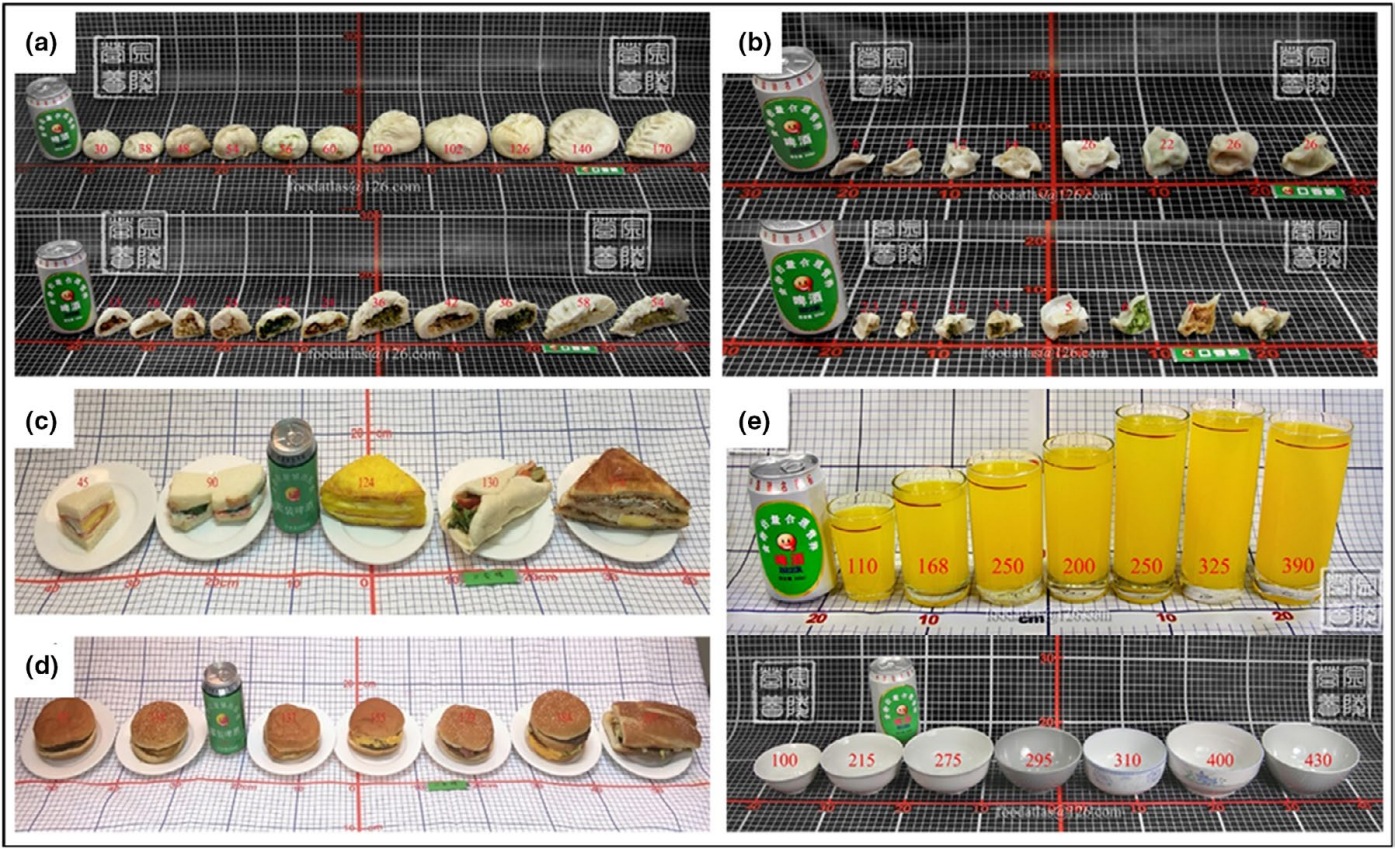
Photos of Chinese and Western representative compound processed foods and tableware. (a) Steamed stuffed buns. (b) Dumplings. (c) Sandwiches. (d) Hamburgers. (e) Glasses (above) and bowls (below) of different sizes.

Compound processed food is composed of a variety of food materials. As a result of differences in eating habits, the food materials in these foods are often inconsistent in each region of China. Therefore, some classic food materials were selected. The compound processed foods shown in the atlas include not only food that represented Chinese cuisine (such as dumplings, glutinous rice balls, steamed stuffed bun, wontons, rice dumplings, eggplant boxes and fried radish balls), but also featured Western foods (such as sandwiches, pizzas and hamburgers). The pictures show dumplings, steamed stuffed buns, sandwiches and hamburgers as examples (Figure 5).

All foods are displayed on a flat surface with different fillings, different shapes and sizes, and different raw and cooked states. As a result of the complexity of food filling, information about the filling and the specific processing methods is added in the text.

#### Tableware

The glasses and bowls commonly used in Chinese families were selected and placed in the order from small to large (Figure 5e). The number marked in the picture with glass cups is the liquid capacity (mL) to the scale at the top of the glass; the number marked in the picture with the bowls is the liquid capacity (mL) to 0.5 cm from the mouth of the bowl.

### Validation of the food atlas

Means and SDs of the estimated weight before and after using the food atlas are summarised, together with the actual weight, in Table 2. The results showed that, except for mushrooms and pork, the mean value of food weight estimated with the auxiliary reference food atlas was closer to the actual weight than that estimated without the food atlas. Furthermore, the variation range of the estimated weight of all foods after using the atlas was smaller and more stable than that estimated without the food atlas.

**Table 2:** Amount of food estimated with and without the auxiliary reference food atlas

Food name	Actual weight	Estimated weight (before)	Estimated weight (after)
	(g)	(g), mean (SD)	(g), mean (SD)
Rice	98	78.90 (31.79)	92.80 (13.63)
Tomato	111	89.00 (27.65)	114.50 (22.53)
Celery	98	83.00 (21.57)	94.00 (15.12)
Mushroom	18	17.00 (7.56)	19.00 (3.78)
Rape	137	107.00 (39.14)	126.74 (21.88)
Pork	85	77.50 (39.85)	72.50 (20.98)
Crucian carp	40	37.00 (13.21)	41.50 (4.55)
Egg	85	89.00 (24.41)	87.00 (23.82)
Orange	33	24.20 (11.26)	33.20 (9.35)
Banana	92	67.50 (19.72)	94.20 (12.30)

Before: estimation of food weight without the auxiliary reference food atlas. After: estimation of food weight with the auxiliary reference food atlas.

As shown in Table 3, a further calculation was performed. The estimated *d*‐value of all foods except eggs was <0, indicating that the respondents underestimated the food weight. After using the food atlas, the estimated *d*‐value became smaller and had a positive value, indicating that the rectification of food weight using the food atlas was directional. The absolute value was further taken, and it was found that the means and SDs of *D* obtained with the food atlas were lower than those obtained before referring to the atlas, and the differences between estimation before and after using the food atlas of all foods were significant (*P* < 0.05). Comparing the differences before using the food atlas, the error ranges of these food samples were reduced by 21.13% for rice, 10.9% for tomato, 7.14% for celery, 17.78% for mushroom, 15.11% for rape, 15.30% for pork, 19.75% for crucian carp, 2.35% for egg, 13.33% for orange and 17.28% for banana.

**Table 3:** Comparison of differences of each food estimated with and without using the food atlas

Food name	Relative difference (*d*)						Absolute difference (*D*)					
	*d* (before)	*d* % (before)	*d* (after)	*d* % (after)	*t*	*P*‐value	*D* (before)	*D* % (before)	*D* (after)	*D* % (after)	*t*	*P*‐value
	(g), mean (SD)	(%)	(g), mean (SD)	(%)			(g), mean (SD)	(%)	(g), mean (SD)	(%)		
Rice	−19.10 (31.79)	−19.49%	−5.20 (13.63)	−5.31%	−5.07	0.000	33.10 (16.27)	33.78%	12.40 (7.52)	12.65%	13.54	0.000
Tomato	−22.00 (27.65)	−19.82%	3.50 (22.53)	3.15%	−12.11	0.000	29.80 (18.76)	26.85%	17.70 (14.15)	15.95%	4.10	0.000
Celery	−15.00 (21.57)	−15.31%	−4.00 (15.12)	−4.08%	−6.92	0.000	20.60 (16.18)	21.02%	13.60 (7.49)	13.88%	3.50	0.001
Mushroom	−1.00 (7.56)	−5.56%	1.00 (3.78)	5.56%	−3.50	0.001	6.60 (3.70)	36.67%	3.40 (1.87)	18.89%	10.06	0.000
Rape	−30.00 (39.14)	−21.90%	−10.26 (21.88)	−7.50%	−4.10	0.000	41.80 (25.83)	30.51%	21.10 (11.48)	15.40%	7.09	0.000
Pork	−7.50 (39.85)	−8.82%	−12.50 (20.98)	−14.71%	1.61	0.115	34.50 (20.76)	40.59%	21.50 (11.31)	25.29%	5.53	0.000
Crucian carp	−3.00 (13.21)	−7.5%	1.50 (4.55)	3.75%	−3.32	0.002	11.40 (7.15)	28.50%	3.50 (3.23)	8.75%	7.97	0.000
Egg	4.00 (24.41)	4.71%	2.00 (23.82)	2.35%	3.50	0.001	22.00 (10.88)	25.88%	20.00 (12.78)	23.53%	3.50	0.001
Orange	−8.80 (11.26)	−26.67%	0.20 (9.35)	0.61%	−17.47	0.000	12.20 (7.35)	36.97%	7.80 (5.05)	23.64%	4.49	0.000
Banana	−24.50 (19.72)	−26.63%	2.20 (12.30)	2.39%	−17.31	0.000	27.70 (14.78)	30.11%	11.80 (3.77)	12.83%	6.83	0.000

*d, relative difference; D, absolute difference*.

*d *= estimated weight − actual weight; *D* = |estimated weight − actual weight|.

*d* % = [(estimated weight − actual weight)/real weight] ×100; *D*% = (|estimated weight − actual weight|/real weight) × 100.

before: estimation of food weight without the auxiliary reference food atlas; after: estimation of food weight with the auxiliary reference food atlas.

## DISCUSSION

There have been several studies on portion size estimation aids for Asian foods; however, most are only available for their own local food and dietary habits, excluding China^27^. Thus, we developed and validated the utility of a food atlas for dietary data collection in China. The food atlas that we developed addressed this gap well by adding three visual reference elements: regularly placed food portions, background coordinates and objects common to daily life. With the help of this innovative visual reference system, the main task of which is to train the respondents in advance and use the food atlas in the survey site, respondents can effectively convert their visual impression of food in memory into food weight as accurately as possible. In addition to this most innovative feature, this food atlas has many other advantages. It not only covered most varieties of food in China at present, covering 303 types of food and two types of tableware, but also covered a variety of different states and forms of food, involving different processing and cooking methods. Furthermore, the weight of all types of foods that can be 100% consumed in their classical state was marked in this atlas. These two characteristics can help respondents to accurately and easily judge the intake of various foods. The foods featured in the atlas also had graduated food portions, which represented the ranges of portions that Chinese people usually consumed. Previous food atlases used in different countries and populations had different numbers of food portions, ranging from one to eight^10^. The greater the number of food portions, the smaller the estimation error^28^. The number of food portions in our atlas is greater than five, thus reducing the estimation error. Additionally, our food atlas was combined with the relevant information data of the food, such as the percentage of edible parts, raw/cooked weight ratio and dry/wet weight ratio, as well as the relevant food concept and information about energy and nutrients in food. These characteristics equipped the atlas with the capability to expand nutrition knowledge and education and, importantly, to facilitate and improve the accuracy of dietary data collection.

It is important to determine whether the estimation of food quantity deviates when using the food atlas. In some studies, food atlases were tested during or immediately after serving^6^, ^29^, ^30^. We evaluated the accuracy of processed and cooked food assisted with food pictures from this atlas, which were consistent with others, and the results were also conducive to promotion. Although the food validated in the present study was not representative of all types of the foods in the atlas, we chose those foods commonly consumed in China. Our results showed that the mean and SD of the differences in food estimations were significantly decreased after using the atlas. It is significantly better than estimating the food weight directly without the food atlas. We also found that the relative weight difference before referring to the atlas was negative (except in eggs), which suggested that most people underestimated the weight of food before using the atlas. Furthermore, we found that the error ranges for all foods after referring to the atlas were significantly reduced, which indicated that more accurate evaluation data were obtained after using the newly developed food atlas. In other words, there is less deviation in the estimation of food quantity using this food atlas, which is really the goal we want to achieve.

This atlas also has limitations. It does not contain milk or its products. However, based on the information in this atlas and the current consumption patterns of milk and dairy products in China, it is still possible to estimate the weight of milk. In China, liquid milk, cheese, condensed milk and other dairy products are generally purchased in supermarkets, with accurate weight or volume^31^. Milk powder is a special type of milk product, and the volume after adding water can be estimated according to the volume of the glass (Figure 5e). Furthermore, our food atlas was only used to verify the dietary recall method, which should be extended to the general population and other dietary survey methods such as food frequency.

## CONCLUSIONS

It is hoped that this is the beginning of an effort to make the investigation of dietary intake more feasible and less biased, and also that other researchers will use the atlas to enable a better understanding of dietary intakes and nutritional adequacy in the Chinese population. We have developed programmes to include these food photos in digital tools for dietary data collection. Through these digital platforms, our food atlas can be used for future data collection, especially in the dietary survey of Chinese adults. However, further work is needed to validate the food atlas for use in different Chinese populations (such as children and the elderly) and, consequently, for use in larger‐scale surveys of dietary intake.

## CONFLICT OF INTEREST

The authors declare that they have no competing interests.

## AUTHOR CONTRIBUTIONS

The authors’ responsibilities were as follows‐‐ZW and YD were responsible for the conception and design of this study. SZ and ZC conducted the study on food portions. YD, YY, FL, YS, ZS and CZ prepared the original draft. ZW, YD and YY reviewed and edited the manuscript. ZW, YD, YY, FL and ZS were responsible for photography; YD and YY were responsible for post‐processing and production of food pictures. ZW, YD, YY, FL, YS, PF, ZL, MZ, XL, TJ, CS, DC, XP, LY and YS were responsible for the collection and preparation of food and the implementation of food atlas. ZW had primary responsibility for the final content. All authors read and approved the final manuscript.

## ETHICAL APPROVAL

The project was approved by the Human Research Ethics Committee of Nanjing Medical University (Protocol # 33; 2014/2015).

## TRANSPARENCY DECLARATION

The lead author affirms that this manuscript is an honest, accurate and transparent account of the study being reported. The reporting of this work is compliant with STROBE guidelines. The lead author affirms that no important aspects of the study have been omitted and that any discrepancies from the study as planned have been explained.

## Supporting information

Fig S1–S8
