# Supplementary material for: Development and validation of a photographic atlas of food portions for accurate quantification of dietary intakes in China
Source: J Hum Nutr Diet. 2021 Jan 6;34(3):604–15. doi: 10.1111/jhn.12844 (PMC8246756; doi:10.1111/jhn.12844)
Supplement: Supplementary file 1 — Fig S1–S8 [file JHN-34-604-s001.docx]

Supplementary material


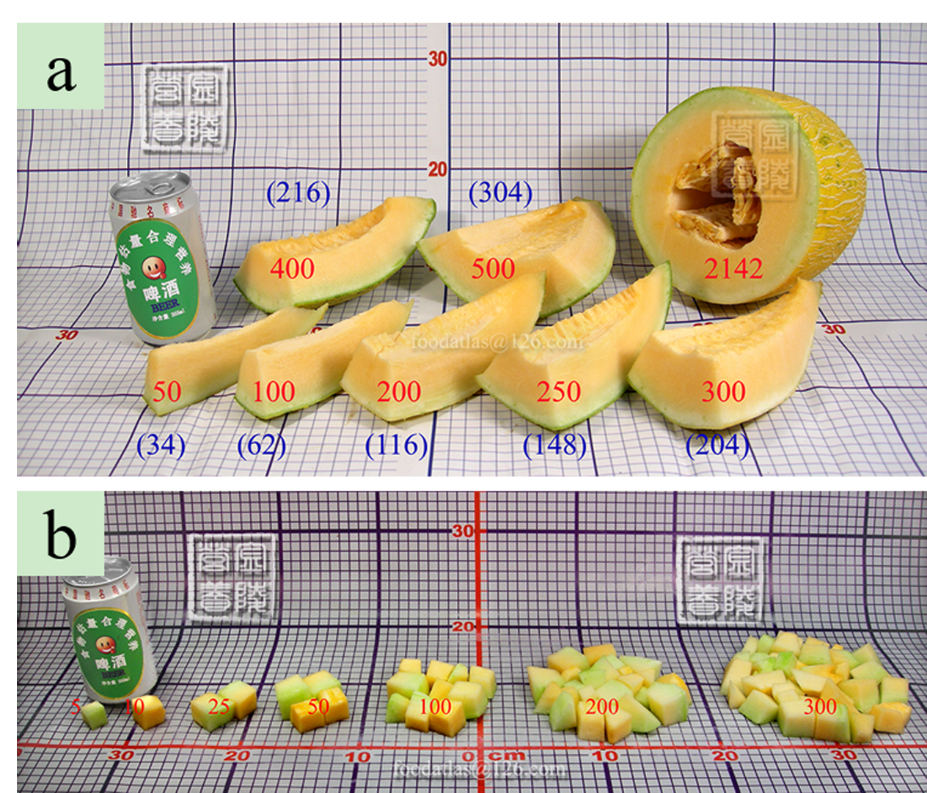


**Figure S1** **Photos of Hami melon.** (a) Hami melon cut into chunks (b) Diced Hami melon


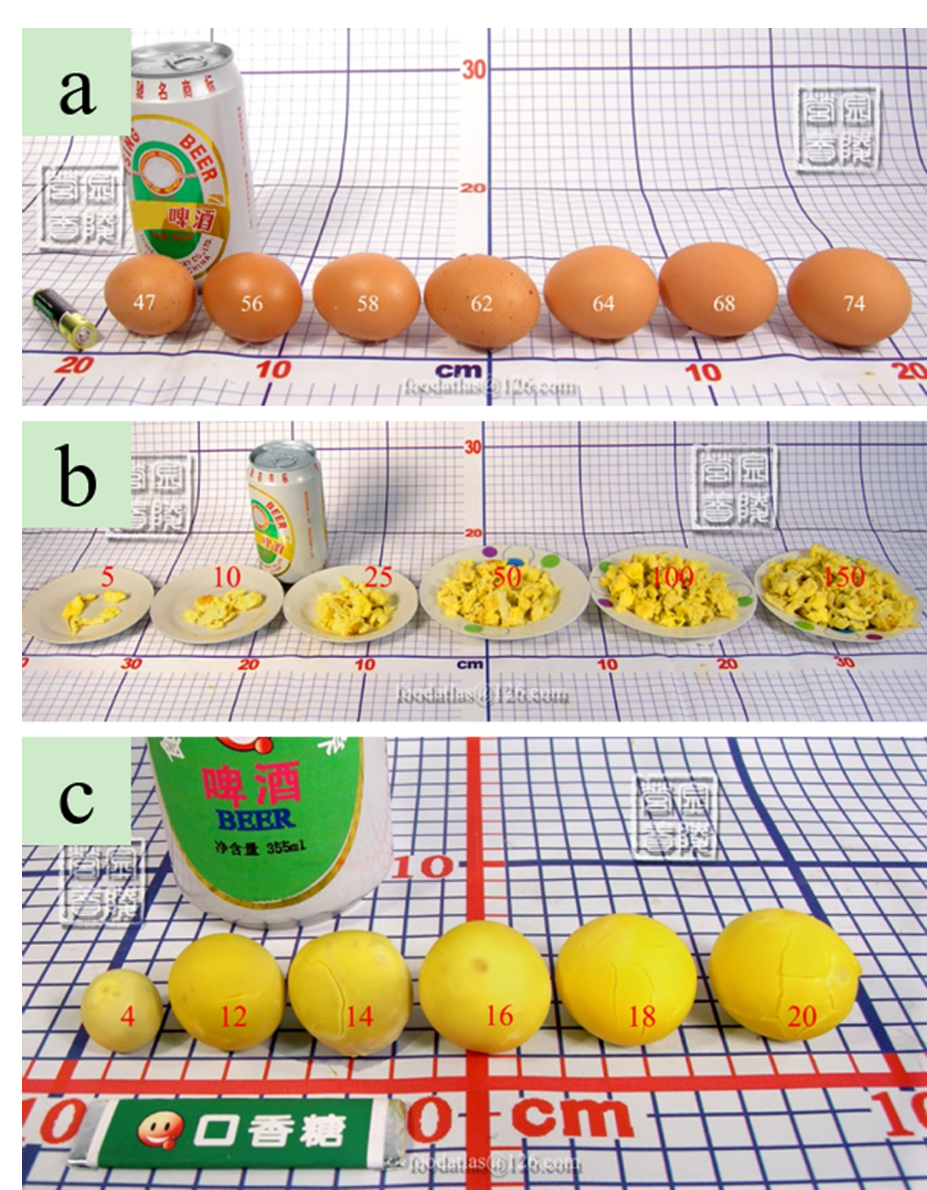


**Figure S2 Photos of eggs.** (a) Chicken eggs in the shell (b) Scrambled eggs (c) Full egg yolks


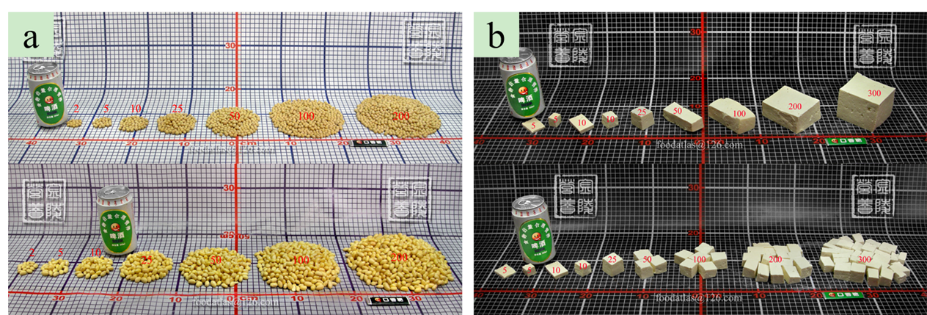


**Figure S3** **Photos of soybeans and soybean products.** (a) Dried (above) and soaked (below) soybeans (b) Whole (above) and divided (below) bean curd


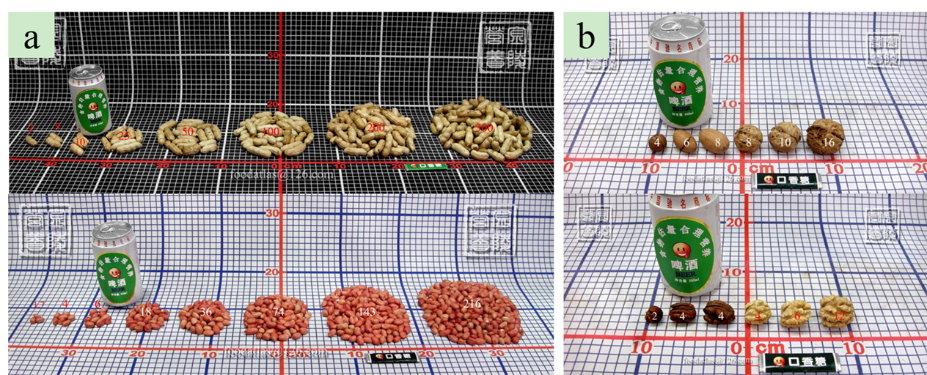


**Figure S4 Photos of nuts.** (a) Peanuts with (above) or without (below) shell (b) Walnuts with (above) or without (below) shell


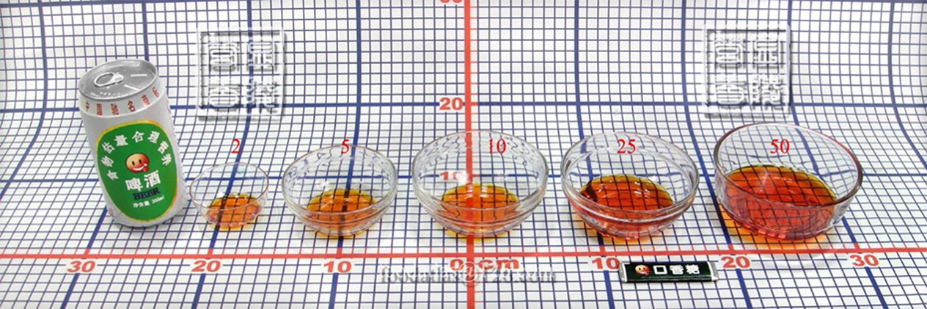


**Figure S5 Photos of chilli oil.**


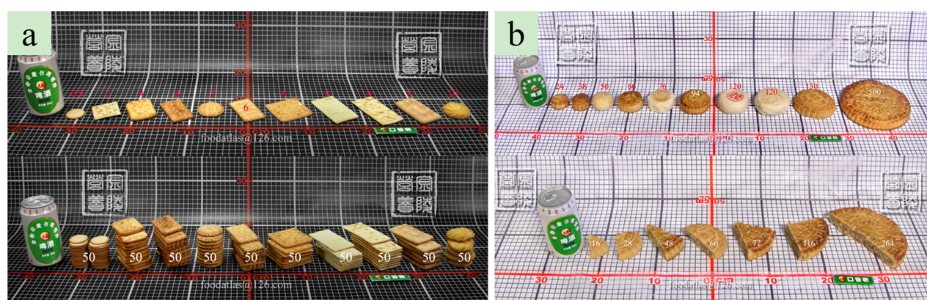


**Figure S6 Photos of cakes.** (a) Biscuits (b) Mooncakes


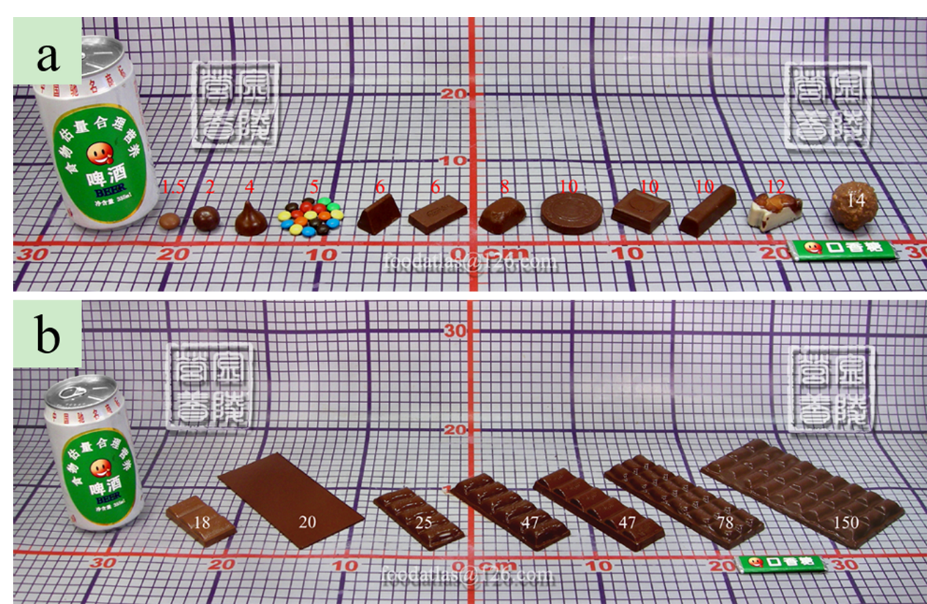


**Figure S7 Photos of chocolates.** (a) Chocolates with different shapes (b) Chocolates with different weights


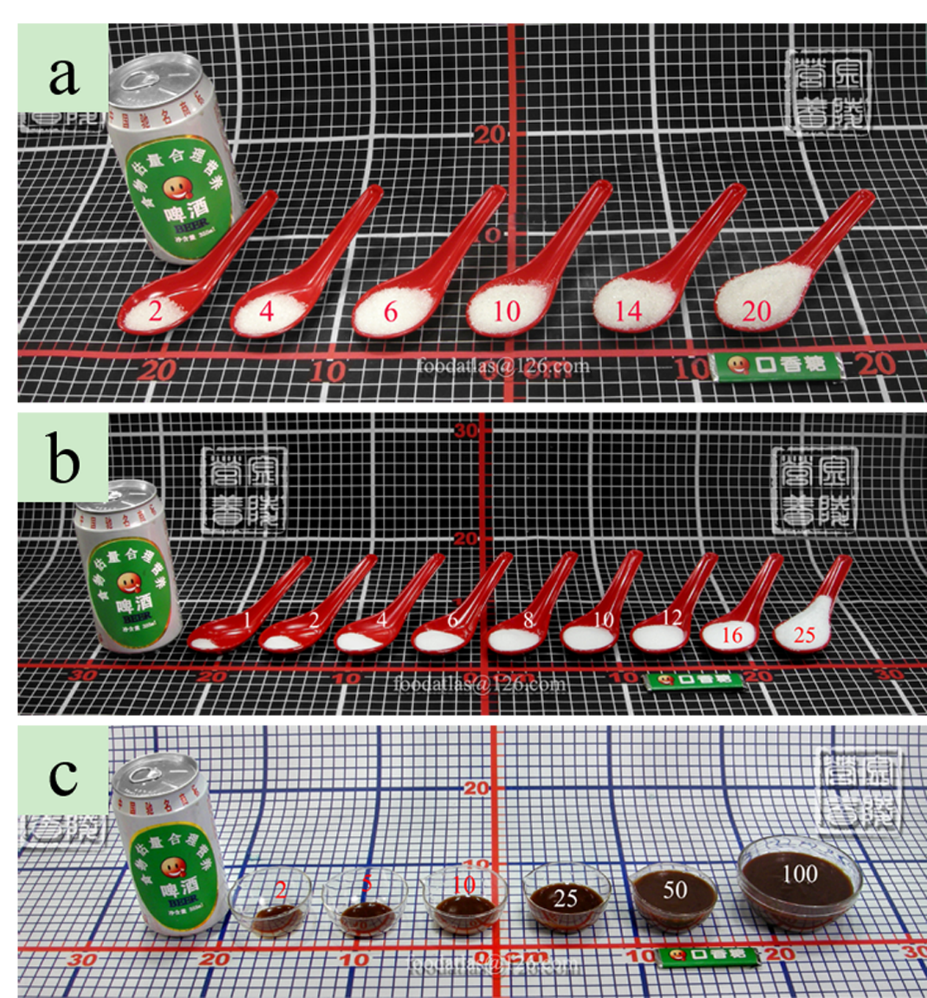


**Figure S8 Photos of condiments.** (a) White sugar (b) Iodized salt (c) Sweet sauce
